# Raltitrexed versus 5‐fluorouracil with cisplatin and concurrent radiotherapy for locally advanced nasopharyngeal carcinoma: An open labeled, randomized, controlled, and multicenter clinical trial

**DOI:** 10.1002/cam4.3260

**Published:** 2020-07-12

**Authors:** Pengwei Yan, Haitao Yin, Wenjie Guo, Xiangdong Sun, Feng Li, Shengfu Huang, Xiuhua Bian, Feijiang Wang, Fuzheng Zhang, Buhai Wang, Hongping Zhou, Chong Zhou, Li Yin, Xuesong Jiang, Ning Jiang, Jianfeng Wu, Juying Liu, Dan Song, Xia He

**Affiliations:** ^1^ Department of Radiaotherapy Jiangsu Cancer Hospital & Jiangsu Institute of Cancer Research The Affiliated Cancer Hospital of Nanjing Medical University Nanjing China; ^2^ Department of Radiotherapy Xuzhou Center Hospital Xuzhou China; ^3^ Department of Radiotherapy Jinling Hospital Nanjing China; ^4^ Department of Radiation Oncology Affiliated Hospital of Jiangnan University Wuxi China; ^5^ Cancer Institute of Northern Jiangsu People's Hospital Yangzhou China; ^6^ Director of Department of Radiotherapy Oncology Nanjing Benq Medicalcenter Nanjing China

**Keywords:** concurrent chemoradiotherapy, nasopharyngeal carcinoma, raltitrexed, tolerability

## Abstract

**Background:**

This study aimed to compare the efficacy and toxicity of raltitrexed (Saiweijian^®^) plus cisplatin (SP regimen) and 5‐fluorouracil plus cisplatin (FP regimen) as concurrent chemoradiotherapy (CCRT) in patients with locally advanced nasopharyngeal carcinoma (LA‐NPC).

**Methods:**

Eligible patients (N = 135) were allocated randomly in a ratio of 1:1 to receive CCRT with either SP or FP. At least 2 cycles of chemotherapy was administrated during radiotherapy. Progression free survival (PFS) was primary endpoint. Secondary endpoints included overall survival (OS), loco‐regional relapse free survival (LRRFS), distant metastasis free survival (DMFS) and toxicity.

**Results:**

In this study, 68 patients received SP as CCRT, and 67 received FP. Objective responses were noted in 97.1% of the patients in the SP group and in 97.0% of the patients in the FP group (*P* = 1.00). At the end of a median 36 months follow‐up period, the estimated 3‐year PFS rates were 70.1% for SP and 66.6% for FP, respectively. The 3‐year LRRFS, DMFS and OS rates were 88.9%, 74.7% and 84.0%, respectively, for the SP group, and 92.3%, 71.0% and 73.7%, respectively, for the FP group. Overall, there was no difference between treatment groups with regard to response or survival. The most frequent acute toxicities monitored in both groups were bone marrow suppression, gastrointestinal side effects and oral mucositis (OM). The overall incidence of grade 3‐4 OM in the FP group (47.8%) was higher than in the SP group (11.8%). However, the incidence of other adverse effects observed in both groups was similar (*P* > .05).

**Conclusions:**

These data indicate that SP and FP therapies have similar efficacy in treating LA‐NPC. The SP regimen showed a tolerable safety profile along with a lower frequency of severe OM and therefore, an improved life quality. In conclusion, SP was a well tolerated, effective, regimen for LA‐NPC treatment.

## Graphical abstract

The incidence of other adverse effects seen in both groups was similar (*P* > .05).The efficacy of both regimens i.e. SP and FP are found similar. The SP regimen has tolerable safety profile, with a lower incidence of severe OM and consequently, an improved quality of life.

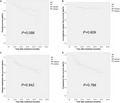

## 1 INTRODUCTION

Nasopharyngeal carcinoma (NPC) is a widespread malignancy of the head and neck in Southeast Asia and Southern China, with approximately new incidences of 50/100 000 per year and the majority of patients were in advanced stage at the time of diagnosis.^1^, ^2^ The standard of locally advanced NPC (LA‐NPC) treatment is concurrent chemoradiotherapy (CCRT), commonly involving 5‐fluorouracil (5‐FU) and cisplatin.^3^, ^4^, ^5^, ^6^, ^7^, ^8^ It was found that 5‐FU has high incidence of causing oral mucositis (OM), leading a higher hospitalization rate and might antagonized with treatment plans.^9^, ^10^

Raltitrexed (Saiweijian^®^), with a similar but not identical mechanism of action with 5‐FU, had been reported to have a good tolerability in the treatment of advanced NPC,^11^, ^12^, ^13^ but had not yet been used for CCRT of LA‐NPC. We have designed this prospective study to postulate whether raltitrexed (Saiweijian^®^)/cisplatin (SP) regimen is more efficacious with reduced adverse events in comparison with FP. The purpose of this study was to inspect the clinical results and toxicity between SP and FP in LA‐NPC patients who treated with CCRT.

## 2 MATERIALS AND METHODS

This randomized, open‐label, controlled study was performed according to the Declaration of Helsinki. The study protocol was accepted by ethics committee of Jiangsu Cancer Hospital, and all patients were given the informed consent in written form.

### 2.1 Patients

Eligible patients met the inclusion criteria as following: (a) age was ranged between 18 and 70 years; (b) pathologically diagnosed as differentiated nonkeratinizing carcinoma or undifferentiated nonkeratinizing carcinoma (WHO II or III); (c) staged T3‐4N0‐3M0 or T1‐4N1‐3M0 (The 7th edition American Joint Committee on Cancer staging system); (d) adequate hematological function, renal function and hepatic function; (e) a performance status as Karnofsky ≥70. The exclusion criteria were: (a) previous received chemotherapy or radiotherapy for NPC; (b) previous diagnosed as a second malignant tumor; (c) the presence of uncontrolled life‐threatening illness; (d) pregnancy or lactation.

### 2.2 Randomization and masking

A ratio of 1:1 was assigned to all eligible patients randomly to receive either SP or FP with a six block size (only known to the statistician). A computer‐generated random code was used for random assignment at Clinical Trials Centre of Jiangsu Cancer Hospital. The details of the random allocations were contained in sequentially numbered, opaque, sealed envelopes prepared by a statistician, who was also involved in the statistical analysis. Treatment assignments were unmasked to clinicians and patients. After the informed consent was collected from eligible patients, the investigators opened the envelopes sequentially and allocated patients to the corresponding interventions.

### 2.3 Procedures

Pretreatment assessment included a detailed physical examination, fiber optic nasopharyngoscopy, enhanced magnetic resonance imaging (MRI) of neck and head, computed tomography (CT) scan of chest and upper abdomen, bone scan, blood cell analysis, biochemical profile, electrocardiography, plasma Epstein‐Barr virus (EBV) DNA load measured by quantitative PCR, and EBV serology at baseline. Whole body ^18^F‐fluorodeoxyglucose (^18^F‐FDG) positron emission tomography (PET‐CT) was optional and was performed at the discretion of the attending physician.

### 2.4 Radiotherapy

All patients received intensity‐modulated radiotherapy (IMRT). We used thermoplastic masks to immobilize patients in supine position before treatment. Intravenous enhanced positioning CT scans (3 mm slices from the head to 2 cm below the sternoclavicular joints) was performed for planning. The prescribed doses were 66‐72 Gy/31‐35 fractions to the planning target volume (PTV) of primary gross tumor volume (GTVnx), 64‐70 Gy/28‐33 fractions to the PTV of involved lymph nodes volume (GTVnd), 56‐60 Gy/28‐30 fractions to the PTV of high‐risk clinical target volume (CTV1), and 50‐54 Gy/28‐30 fractions to the PTV of low‐risk clinical target volume (CTV2). A boost to PTV of GTVnx and GTVnd was delivered for patients with locally or regionally residual tumor after prescribed dose.

### 2.5 Chemotherapy

SP group was treated with raltitrexed ([Saiweijian®], 2.5 mg/m^2^ intravenous infusion on day 1) and cisplatin (25 mg/m^2^ daily intravenous infusion on day 1‐3) at an interval of 3 weeks from the first day as IMRT started. FP group was treated with 5‐FU (800 mg/ m^2^ daily on day 1‐5 as a 120‐hours intravenous infusion) and cisplatin (25 mg/m^2^ daily intravenous infusion on day 1‐3) at an interval of 3 weeks from the first day as IMRT started.

### 2.6 Patient assessment and follow up

Severe and late adverse cases were evaluated in accordance with the Common Terminology Criteria for Adverse Events v3.0 protocol. Patients’ health status was weekly monitored during CCRT. The follow‐up was counted from the day of first treatment to the day of last examination or death. Patients were followed every three months in first three years, every six months for the fourth and fifth years, and every year afterward. The follow‐up examinations included complaints query, physical examinations, MRI or CT of the head and neck region, chest CT scan, upper abdominal CT scan or ultrasound, bone scan, EBV DNA load, and EBV serological testing. PET‐CT scans were conducted when clinically indicated. The primary endpoint was progression‐free survival (PFS, the time from the first day of treatment to documented local or regional relapse, distant metastasis, or death from any cause, whichever occurred first) at 3 years and the secondary endpoints included overall survival (OS, time to death from any reason), loco‐regional relapse‐free survival (LRRFS, time to local or regional recurrence or both), distant metastasis‐free survival (DMFS, time to distant metastasis) and toxicity.

### 2.7 Statistical analysis

The statistical software SPSS 19 was used (SPSS Inc.). Comparison of occurrence rates of adverse events and categorical variables were analyzed by Chi‐squared test. The Kaplan‐Meier method was used for survival analysis and the log‐rank test was used to compare difference. Two‐sided *P *< .05 were considered as significant.

## 3 RESULTS

Between November 2014 and May 2017, 139 eligible patients were randomly assigned to receive SP (n = 69) and FP (n = 70) across six sites in Jiangsu Province China. One patient assigned to SP group and three patients assigned to FP group withdrew consent before the allocated treatment. Among all the 139 patients, 102 were males and 33 were females. The median age was 56 years, and 27 (19.4%) patients received PET‐CT for staging before treatment. There was no significant differences found between the two groups in baseline characteristics (Table 1). All these 139 patients completed IMRT and two cycles of concurrent chemotherapy. Notably, there were 4 patients in the FP group received boost of PTV of GTVnx and GTVnd, while none in the SP group needed this boost. Three months after CCRT, the objective response rates (CR plus PR) were 97.1% (66/68) in SP group and 97.0% (65/67) in FP group (*P* = 1.000). The locoregional or distant failure patterns in both groups were found similar (Table 2).

**TABLE 1:** Characteristics of patients with LA‐NPC

Characteristics	SP (cases [%])	FP (cases [%])	*χ* ^2^	*P*
Total	68	67		
Age (y)				
<50	19 (27.9)	27 (40.3)	2.294	.130
≥50	49 (72.1)	40 (59.7)		
Gender				
Male	51 (75.0)	51 (76.1)	0.023	.880
Female	17 (25.0)	16 (23.9)		
T stage				
T1	2 (2.9)	3 (4.5)	1.541	.673
T2	21 (30.8)	15 (22.3)		
T3	33 (48.5)	34 (50.7)		
T4	12 (17.6)	15 (22.3)		
N stage				
N0	4 (5.8)	4 (5.9)	0.387	.943
N1	28 (41.2)	25 (37.3)		
N2	29 (42.6)	32 (47.8)		
N3	7 (10.3)	6 (8.9)		
Clinical stage				
III	42 (61.8)	43 (64.2)	0.166	.683
IV	26 (38.2)	24 (35.8)		
WHO histology				
II	8 (11.8)	7 (10.4)	0.059	.808
III	60 (88.2)	60 (89.6)		

Abbreviations: FP, 5‐fluorouracil/cisplatin; IMRT, intensity‐modulated radiotherapy; SP, raltitrexed/cisplatin; WHO, World Health Organization.

**TABLE 2:** Patterns of disease failure in patients treated with SP vs FP

Failure pattern	SP (cases [%])	FP (cases [%])	*P*
Locoregional relapse only	4 (5.9)	4 (5.9)	.983
Distant metastases only	16 (23.5)	17 (25.4)	.803
Both locoregional relapse and distant metastases	4 (5.9)	3 (4.5)	.713
Death	11 (16.2)	19 (28.4)	.089

Abbreviations: FP, 5‐fluorouracil/cisplatin; SP, raltitrexed/cisplatin.

Tolerably acute toxicities during radiotherapy were observed from the entire cohort, excepted mucositis in the FP group. The SP group had significantly reduced occurrence rates of grades 3‐4 mucositis (11.8% vs 47.8%, *P* < .001) compared with the FP group. A total of 22 patients underwent nasogastric tube insertion or gastrostomy due to oropharyngeal severe pain and swallowing difficulty caused by OM, and all the 22 cases were from the FP group (32.8%). Additionally, 14 cases treated by FP experienced one to two weeks prolongation of CCRT because of intolerable to the original plan. No significant differences in leukopenia, anemia, thrombocytopenia, hepatotoxicity, and nausea‐vomiting were found between the two groups (Table 3). There was no significant difference in late toxicities found between the two groups, and the most common late toxicities were xerostomia, hear loss, skin dystrophy, subcutaneous fibrosis, and temporal lobe injury.

**TABLE 3:** Treatment‐related toxicities monitored in patients with LA‐NPC

Toxicity	SP (cases [%])	FP (cases [%])	*χ* ^2^	*P*
Grade 3/4 acute toxicities				
Leukopenia	6 (8.8)	9 (13.4)	0.780	.377
Anemia	3 (4.4)	2 (3.0)	0.193	.661
Thrombocytopenia	2 (2.9)	3 (4.5)	0.223	.636
Hepatotoxicity	5 (7.4）	2 (3.0)	1.310	.252
Nausea‐vomiting	2 (2.9)	5 (7.5)	1.403	.236
Mucositis	8 (11.8)	32 (47.8)	20.973	<.001
Late toxicities				
Skin dystrophy	23 (33.8)	28 (41.8）	0.911	.340
Subcutaneous fibrosis	14 (20.6)	15 (22.3)	0.065	.799
Xerostomia	39 (57.4)	45 (67.1)	1.382	.240
Hear loss	31 (45.6)	34 (50.7)	0.360	.549
Temporal lobe injury	2 (2.9)	4 (5.9)	0.729	.393

**FIGURE 1:**
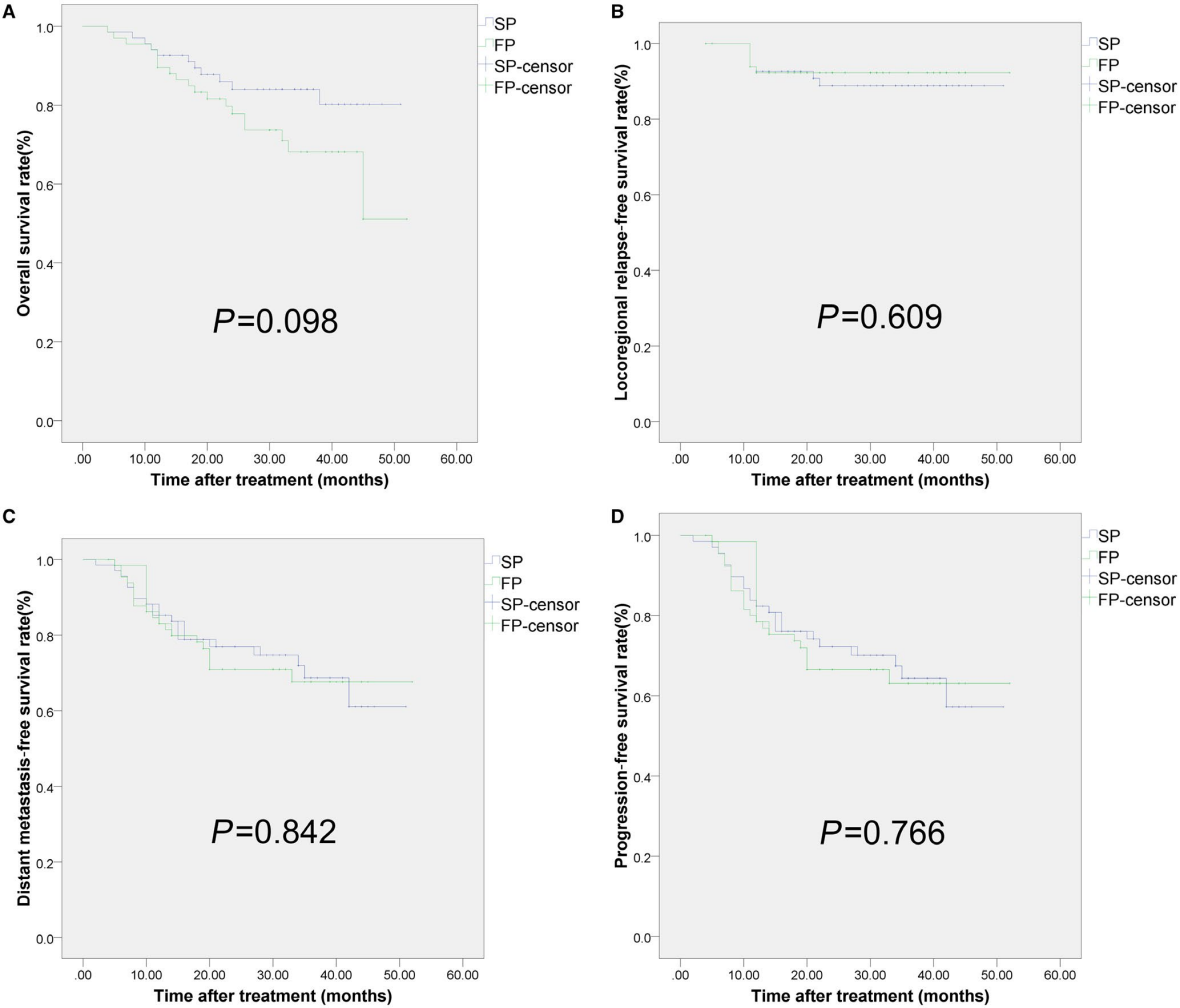
Kaplan–Meier assessments of patients survival with LA‐NPC treated by IMRT with concurrent SP vs FP. A, Overall survival. B, Loco‐regional relapse‐free survival. C, Distant metastasis‐free survival. D, Progression‐free survival. There was no significant difference observed in 3‐year survival rates between the two groups

The estimated 3 year rates of OS, DMFS, LRRFS, and PFS were 84.0, 74.7, 88.9, and 70.1%, respectively, for the SP group, and 73.7, 71.0, 92.3, and 66.6%, respectively, for the FP group. No significant differences were found in the survival rate among two groups (Figure 1).

## 4 DISCUSSION

To the best of our knowledge, this is the first study to investigate the SP regimen and FP regimen in untreated LA‐NPC patients. In the current study, both two regimens yielded favorable clinical outcomes. However, SP regimen was discovered with reduced severe OM compared to FP regimen. Therefore, patients treated with SP were found to have improved quality of life as compared to the patients who were treated with FP.

Of note, IMRT had replaced two‐dimensional radiotherapy and become a principal treatment method for NPC patients.^14^ Because IMRT promoted targeted volume closer to tumor shape, and reduced injury to normal tissues, the local and regional tumor control had a better efficiency in NPC patients. However, as most untreated patients were diagnosed with locally advanced disease, the main failure of NPC had shifted to distant metastasis.^15^, ^16^, ^17^ As a treatment focused on primary gross and cervical lymph nodes, single application of radiotherapy could not improve DMFS. Hopefully, a combination of chemotherapy and radiotherapy had been a standard treatment strategy for LA‐NPC. To improve outcome to this malignancy, different chemotherapy regimens were studied for decades. Non‐platinum‐based regimens manifested inferior survival as compared with cisplatin‐based or other platinum‐based regimens.^18^ As a result, platinum‐based regimens were strongly recommended to treat LA‐NPC. Several centers established single cisplatin in CCRT regimen for clinical application and exploratory research for LA‐NPC.^19^, ^20^ Zhang et al designed a randomized trial to compare induction chemotherapy (IC) plus CCRT with CCRT alone for LA‐NPC. The 3‐year recurrence‐free survival and OS was 76.5% and 90.3% in the standard‐therapy group (administrated with singe cisplatin as CCRT without IC).^20^ Similarly, according to Sun's data, 3‐year failure‐free survival was 72% in single cisplatin‐based CCRT alone group.^19^ Both studies suggested that significantly higher incidence of acute adverse events of grade 3 or 4 was observed in IC + CCRT group than in CCRT group. In our institution, one prior study using singe cisplatin as CCRT for LA‐NPC showed 3‐year PFS and OS were 71.9% and 85.4%, respectively.^21^ Currently, we administrated SP or FP in CCRT and discovered rather close outcome on 3‐year PFS and OS. Notably, our cohort contained more old patients because wider range of age met our inclusion criteria, and more than half of our patients were staged N2‐3 with higher risk of failure.

To avoid prolonging the duration of treatment and increasing the number of hospitalizations, we were not planning to bring IC or adjuvant chemotherapy (AC) into our study before designing. Because a combination of cisplatin with 5‐FU based on past experiences was also frequently used,^22^ we compared SP with FP rather than with cisplatin alone. 5‐FU, an antimetabolite medication that act as a anti‐pyrimidine by impeding DNA synthesis by reticence of enzyme thymidylate synthase (TS),^23^ could induce and aggravate OM during CCRT,^24^, ^25^, ^26^ and would result in extension of treatment plan along with decrease in drug dosage, restriction on the success of cancer chemotherapy, suspension of the treatment, and finally a decrease in survival rate.^25^, ^26^ Injuries related to OM cause severe pain, difficulty swallowing and speech and reduced quality of patient life. Moreover it affects the patient with a greater risk of systemic and local contamination and delays the ability to give the planned dosage of cancer therapy, thus giving a threat to life of patient and increasing the hospital stay of patient as well.^27^ Similarly to current founding, higher grade 3/4 mucositis rate (55%) during CCRT was observed from FP regimen than high‐dose cisplatin tri‐weekly and low‐dose cisplatin (29%‐48%) weekly.^5^, ^28^, ^29^ Additionally, 5‐FU was considered as second most commonly used chemotherapeutic medicine linked with cardiotoxicity after anthracyclines, that can result in acute coronary, chest pain, myocardial syndrome infarction and death.^30^, ^31^ Fluoropyrimidine‐induced cardiotoxicity (FIC), an infrequent but potentially life‐threatening toxicity, should not be ignored but unfortunately remained lack concerning amongst clinicians.^30^, ^31^ Therefore, to improve the chemotherapy regimen, it was necessary to select appropriate drugs to replace 5‐FU rather than cisplatin.

Raltitrexed, an antimetabolic folate analogue that inhibits TS directly and specifically, had a similar but not identical mechanism to 5‐FU. Raltitrexed‐indueced polyglutamate could enhance the anti‐tumor activity of TS by enhancing the inhibition ability and prolonging the inhibition time. Therefore, differently from 5‐FU, raltitrexed was not leucovorin‐depended as bolus injections for administration, and different mechanisms between raltitrexed and 5‐FU might explain the reason why severe OM rates met significant difference. In addition, the T_1/2_ value of raltitrexed was longer than that of 5‐FU, and the effective blood concentration could be maintained without continuous intravenous administration. This implied that raltitrexed could reduce the hospital stay period, serving to resolve the problem of lack of hospital beds in China. FIC was not observed in SP nor FP group, partly because of the limitations of our inclusion and exclusion criteria. However, several studies had proved raltitrexed as a safe medication to heart and could be an alternative to 5‐FU in cancer patients with cardiac history.

Raltitrexed had been reported to have a good tolerance in the treatment of advanced NPC,^11^, ^12^, ^13^ but had not yet been used for CCRT of LA‐NPC. So we designed this prospective and multicenter study to compare the different clinical outcome between SP and FP in LA‐NPC patients. In the current study, patients treated with SP were found to have improved quality of life as compared to the patients who were treated with FP, possibly due to the decrease in indicative adverse events. All the patients medicated with FP described a higher incidence of severe OM, that could be defined by the toxicity related with 5‐FU.^24^, ^25^, ^26^ Additionally, our study suggested SP regimen with reduced nausea–vomiting and xerostomia. Although slightly more patients had grade 3‐4 anemia and hepatotoxicity in the SP group than in the FP group, the rate of grade three or four neutropenia and leucopenia did not vary significantly between two groups. The estimated 3‐year survival rates and failure patterns between two groups were similar, while SP regimen suggested a trend in better response rate and 3‐year OS.

We acknowledge some limitations exist during this study. First, our sample size was not large. Second, the follow‐up period was limited, and individual recruitment spanned long time range. Third, due to the controversy of efficacy, IC or AC combined with CCRT were excluded from treatment. Fourth, because of limited size of sample, this study was unable to perform further subgroup analysis of relevant factors. Finally, this study can be further extended by applying the results to non‐Asian patients.

## 5 CONCLUSION

Our finding suggested that raltitrexed plus cisplatin can be used as an alternative treatment strategy to 5‐FU plus cisplatin as CCRT treatment in the patients with LA‐NPC. Further follow‐up and randomized clinical trials bringing IC or AC into complex treatment are required to establish an effective combination of IMRT and raltitrexed/cisplatin chemotherapy to develop the prediction of patients with LA‐NPC.

## CONFLICTS OF INTEREST

There is no conflict of interest statement.

## Data Availability

The datasets used and/or analyzed during the current study are available from the corresponding author on reasonable request.
